# Omics for understanding the tolerant mechanism of *Trichoderma asperellum* TJ01 to organophosphorus pesticide dichlorvos

**DOI:** 10.1186/s12864-018-4960-y

**Published:** 2018-08-08

**Authors:** Qiong Wu, Mi Ni, Guisheng Wang, Qianqian Liu, Meixia Yu, Jun Tang

**Affiliations:** Anhui Province Key Laboratory of Embryo Development and Reproductive Regulation, Anhui Province Key Laboratory of Environmental Hormone and Reproduction, Fuyang Normal University, Fuyang, 236037 Anhui China

**Keywords:** *Trichoderma asperellum*, Dichlorvos, Transcriptome, Metabolome, Tolerance mechanism

## Abstract

**Backgroud:**

Though it is toxic to humans, dichlorvos is a widely used chemical pesticide and plays an important role in the control of plant pests. The application of a combination of the biocontrol agent *Trichoderma* with dichlorvos may reduce the need for chemical pesticides. Therefore, revealing the specific molecular mechanism of *Trichoderma* tolerance to dichlorvos has become particularly important.

**Results:**

In this study, using transcriptome and metabolome analyses, changes in primary and secondary metabolisms in *Trichoderma asperellum* TJ01 were comprehensively studied in the presence of dichlorvos. A novel C_2_H_2_ zinc finger protein gene, zinc finger chimera 1 (*zfc1*)*,* was discovered to be upregulated, along with a large number of oxidoreductase genes and ABC transporter genes under dichlorvos stress. In addition, gas chromatography-mass spectrometry (GC-TOF-MS), and liquid chromatography-mass spectrometry (LC-QQQ-MS) data revealed the global primary and secondary metabolic changes that occur in *T. asperellum* TJ01 under dichlorvos stress.

**Conclusions:**

The tolerance mechanism of *T. asperellum* TJ01 to dichlorvos was proposed. In addition, the absorption and residue of dichlorvos were analyzed, laying the foundation for elucidation of the mechanism by which *T. asperellum* TJ01 degrades pesticide residues.

**Electronic supplementary material:**

The online version of this article (10.1186/s12864-018-4960-y) contains supplementary material, which is available to authorized users.

## Background

Dichlorvos is an important organic phosphorus pesticide with fast-acting and broad-spectrum activity that has been in use in agricultural production for more than 50 years. However, it causes severe pollution of soil and water [1, 2]. The strong toxicity in non-target organisms poses a considerable threat to the environment and to humans [3]. Therefore, controlling pests with biological agents has garnered much attention in the field of plant protection.

*Trichoderma* is the most widely used and studied fungus in the biological control field. It offers the advantages of high activity and environmental safety. However, due to the limitations of its slow start-up and short shelf life, among others, *Trichoderma* cannot be used to completely replace chemical pesticides. At present, the ideal control strategy may be a combination of *Trichoderma* and dichlorvos, which would allow for a reduction in the amount of pesticides used while achieving the desired control effect [4]. Additionally, *Trichoderma* may contribute to the degradation of dichlorvos residues in the environment. As the tolerance of *Trichoderma* to dichlorvos directly affects the control afforded by the combination of the two pesticides, it is particularly important to investigate the mechanisms of *Trichoderma* tolerance to dichlorvos.

Until now, the tolerance mechanisms of *Trichoderma* to chemical pesticides have only been studied at the physiological and biochemical levels. These results have led to two hypotheses regarding the mechanisms of *Trichoderma* tolerance. The first is that *Trichoderma* is not recognized by chemical pesticides due to changes at target sites. For example, the main action of the fungicide benzimidazole is to bind the β-tubulin of a plant pathogen, thereby inhibiting its mitosis and morphogenesis. *Trichoderma viride* exhibits reduced affinity to pesticides due to mutations in the tubulin protein, resulting in its ability to tolerate benzimidazoles [5]. The second hypothesis is that *Trichoderma* effectively metabolizes or degrades some chemical pesticides. It was discovered that *Trichoderma harzianum* could degrade organochlorine insecticides via its oxidation system [6].

Transcription factors are important players in fungal tolerance to chemical pesticides and pharmaceutical drugs. In *Saccharomyces cerevisiae*, the zinc finger protein transcription factor pleiotropic drug resistance 1 (Pdr1) and its homologous protein Pdr3 are directly involved in the pleiotropic drug response and activate and suppress the expression of anti-drug-related genes, respectively. These regulators of drug-resistance-related genes include multiple ATP binding cassette (ABC) transporter genes, such as *pdr5*, *pdr10,* and *pdr15* [7–9]. In *Aspergillus fumigatus*, the Zn2-Cys6 ABC-transporter regulating transcription factor (AtrR) provides resistance against azoles by regulating the expression of the drug target gene, *Cytochrome P450 Family 51 Subfamily A* (*cyp51A*) and the ABC transporter gene *cdr1B* [10]. In summary, transcription factors may regulate ABC transporter proteins for the removal of harmful substances from drug-resistant fungi and may also control the expression of the target protein recognized by the drug.

In this study, *T. asperellum* TJ01 was selected as it rapid growth, and high inhibition effect and tolerance to dichlorvos. The transcriptome and metabolome profiles were featured to study the tolerance mechanisms of *T. asperellum* TJ01 to dichlorvos. Meanwhile, the absorption and degradation of dichlorvos by *T. asperellum* TJ01 were also analyzed. The tolerance mechanisms discussed here should lay the foundation for use of *T. asperellum* TJ01 in the degradation of pesticide residues.

## Results

### Impact of dichlorvos on growth of *T. asperellum* TJ01

**Fig. 1:**
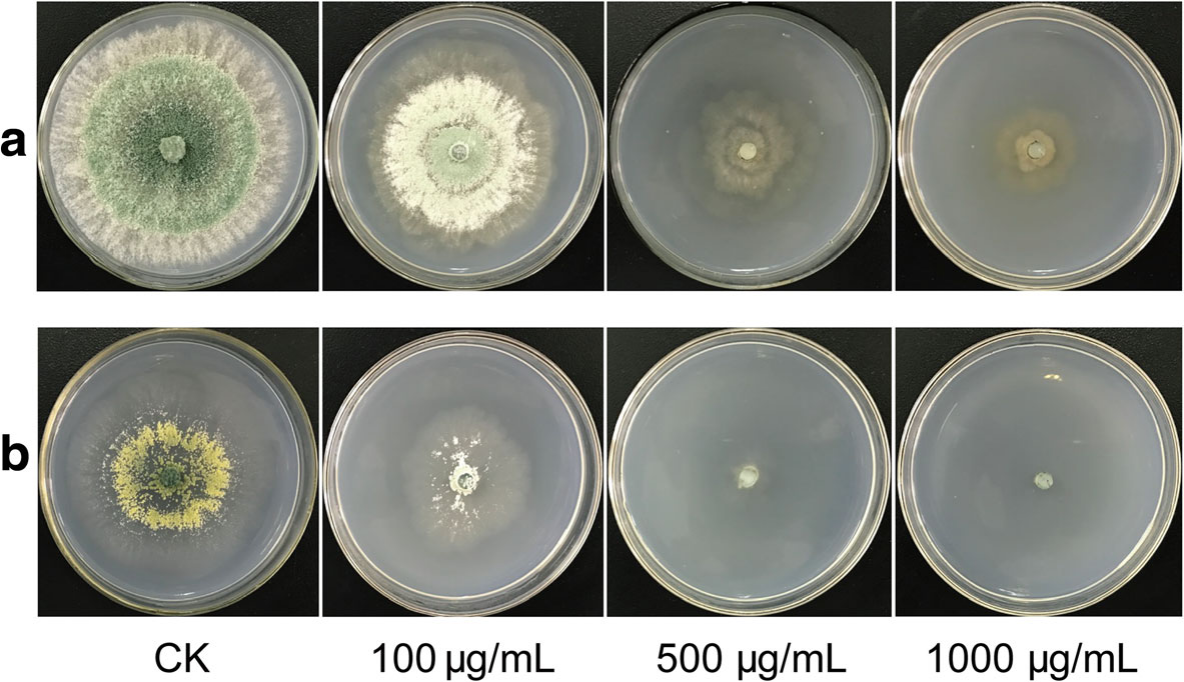
Morphology of *T. asperellum* TJ01 under dichlorvos stress after 3-d cultivation. **a** PDA plate; **b** Burk plate

In this study, we determined the effect of dichlorvos on the growth of *T. asperellum* by solid medium cultrue. After culture on Potato Dextrose Aga (PDA) plates for 3 d, comparing with control, the fungi could grow well when exposured to dichlorvos (100 μg/mL), however, changes in the colony size and mycelium morphology of *T. asperellum* TJ01 were more and more evident as the concentration of dichlorvos increased (Fig. 1a). A nitrogen-poor medium, Burk medium, was also tested for this interaction. Similar tolerance phenotype was observed in a dichlorvos-dependent manner (Fig. 1b).

### Global impact of dichlorvos on *T. asperellum* TJ01

**Fig. 2:**
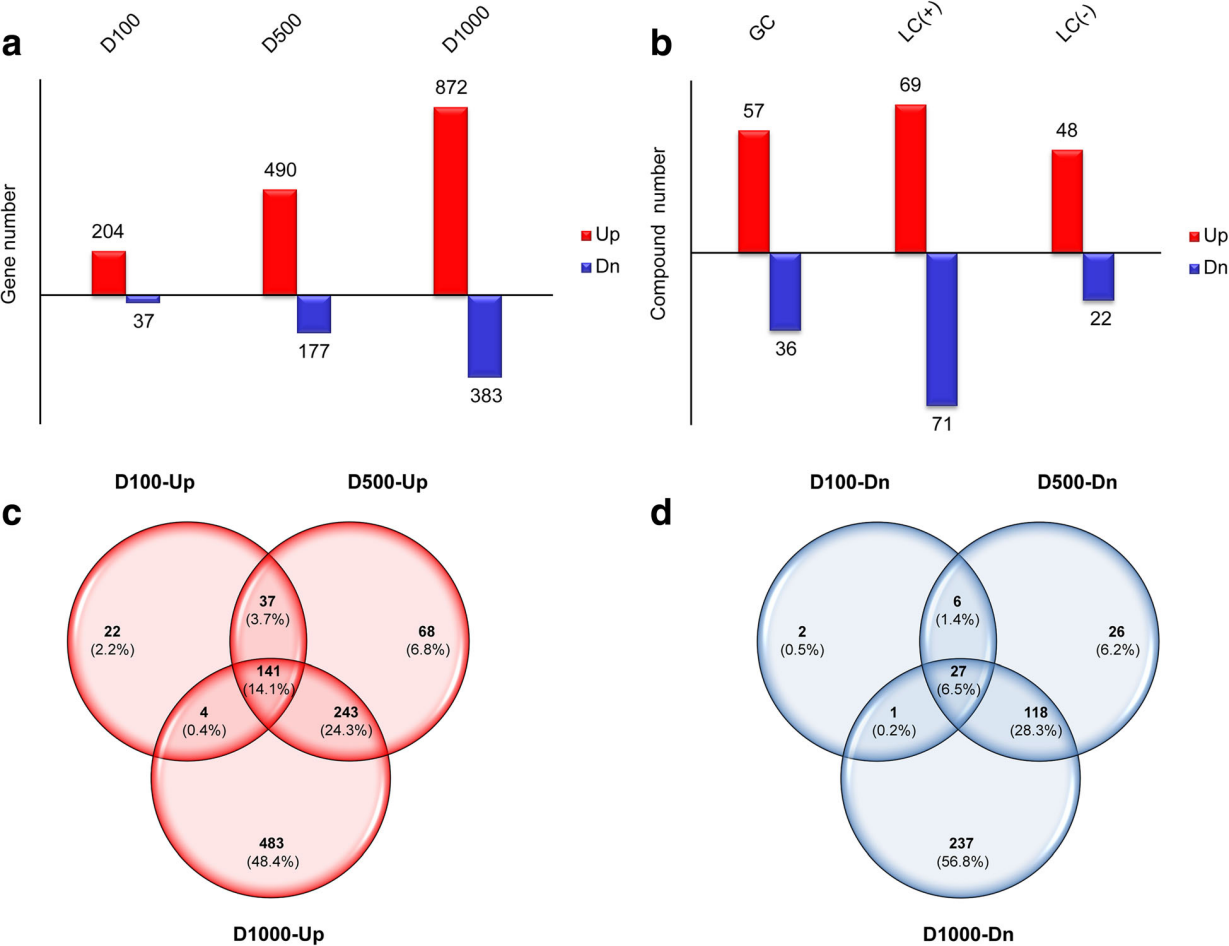
Global impact of dichlorvos on *T. asperellum* TJ01 based on multiomics analysis*.* **a** Number of significantly differentially expressed genes based on RNA-seq analysis of *T. asperellum* TJ01 after treatment with dichlorvos for 24 h. D100: 100 μg/mL dichlorvos treatment/CK; D500: 500 μg/mL dichlorvos treatment/CK; D1000: 1000 μg/mL dichlorvos treatment/CK; **b** Compounds that were significantly altered in *T. asperellum* TJ01 after treatment with 500 μg/mL dichlorvos for 24 h. GC: GC-TOF-MS, LC(+): LC-QQQ-MS(+), LC(−): LC-QQQ-MS(−). Red: significantly upregulated, blue: significantly downregulated; **c**, The relationship of significantly upregulated genes in *T. asperellum* TJ01 treated with 100 μg/mL, 500 μg/mL, and 1000 μg/mL dichlorvos; **d**, The relationship of significantly downregulated genes in *T. asperellum* TJ01 treated with 100 μg/mL, 500 μg/mL, and 1000 μg/mL dichlorvos

Based on RNA-seq analysis, in *T. asperellum* TJ01 treated with 100 μg/mL, 500 μg/mL, and 1000 μg/mL dichlorvos, 204, 490, and 872 genes were significantly upregulated, respectively, while 37, 177, and 383 genes were significantly downregulated, respectively (Fig. 2a).

Following GC-TOF-MS analysis of *T. asperellum* TJ01 for 24 h, 232 compounds were obtained. Compared with the control sample, the sample treated with dichlorvos exhibited 57 compounds that were significantly upregulated and 36 compounds that were significantly downregulated. Following LC-QQQ-MS (+) analysis of *T. asperellum* TJ01 for 24 h, 323 compounds were obtained. Compared with the control sample, the sample treated with dichlorvos treatment exhibited 69 compounds that were significantly upregulated and 71 compounds that were significantly downregulated. Finally, LC-QQQ-MS (−) analysis of *T. asperellum* TJ01 for 24 h resulted in the attainment of 173 compounds. Compared with the control sample, dichlorvos treatment led to significant upregulation of 48 compounds and significant downregulation of 22 compounds (Fig. 2b).

Then, the relationship of significantly changed genes in *T. asperellum* TJ01 treated with 100 μg/mL, 500 μg/mL, and 1000 μg/mL dichlorvos were illustrated by the venn diagram (Fig. 2c and d). While, the compounds detected via GC-TOF-MS, LC-QQQ-MS (+), and LC-QQQ-MS (−) have no coincident.

### Impact of dichlorvos on primary metabolism of *T. asperellum* TJ01

**Fig. 3:**
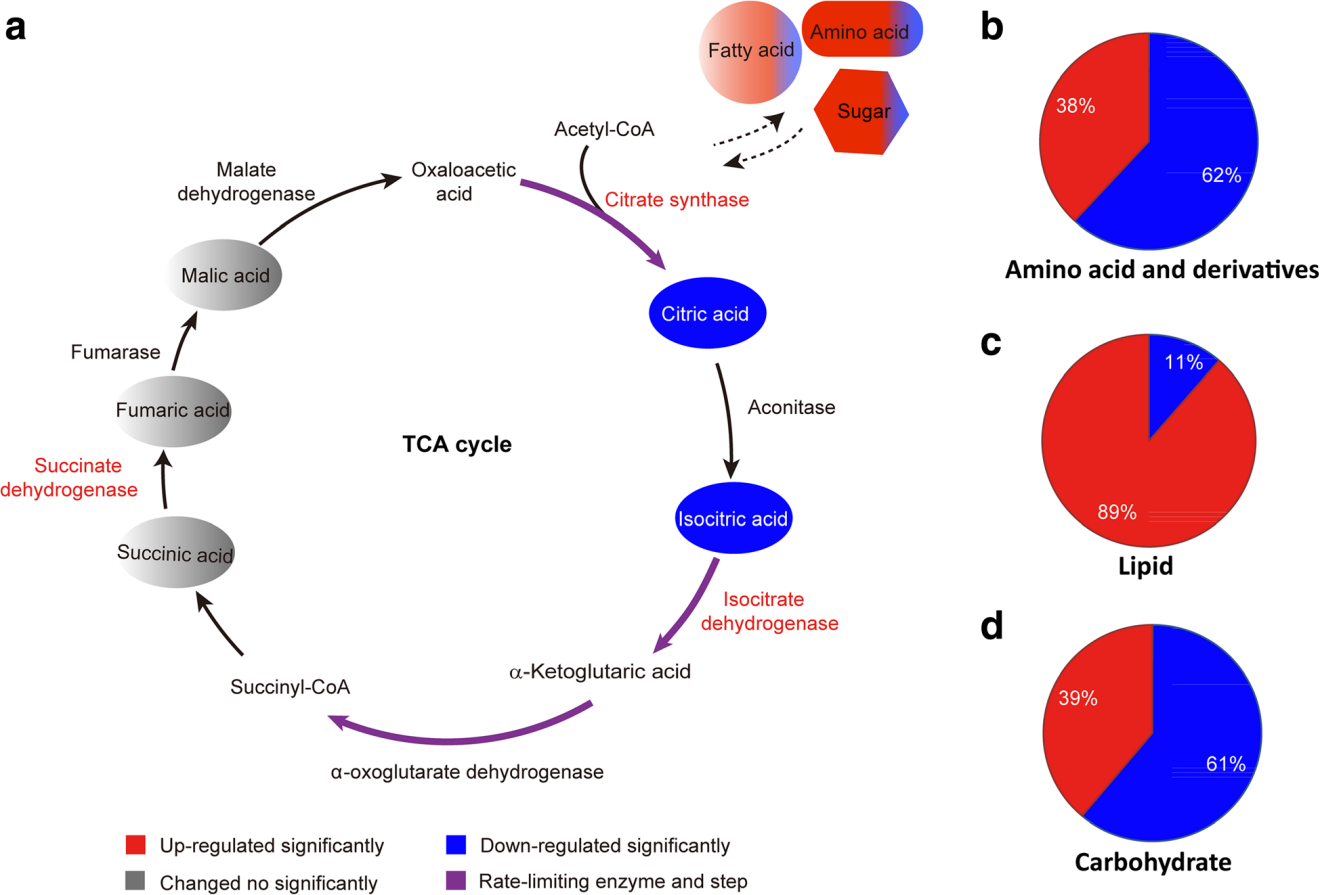
Impact of dichlorvos on primary metabolism of *T. asperellum* TJ01 based on multiomics analysis*.* **a** Impact of dichlorvos on TCA cycle based on RNA-seq, GC-TOF-MS, and LC-QQQ-MS analysis; **b** Impact of dichlorvos on metabolism of amino acids and derivatives based on GC-TOF-MS and LC-QQQ-MS analysis; **c** Impact of dichlorvos on lipid metabolism based on GC-TOF-MS and LC-QQQ-MS analysis; **d** Impact of dichlorvos on carbohydrate metabolism based on GC-TOF-MS and LC-QQQ-MS analysis. Red: significantly upregulated, blue: significantly downregulated, grey: not significantly altered, purple: rate-limiting step

Transcriptome profile analysis of *T. asperellum* TJ01 treated with 500 μg/mL dichlorvos showed that a citrate synthase, isocitrate dehydrogenase, and succinate dehydrogenase were significantly upregulated after treatment with dichlorvos (see Additional file 1). LC-QQQ-MS analysis of *T. asperellum* TJ01 treated with 500 μg/mL dichlorvos showed that citric acid and isocitrate were significantly downregulated, while succinic acid, fumaric acid, and malic acid were not significantly altered. Thus, comprehensive analysis of transcriptome and metabolome data suggested that dichlorvos has obvious effect on the tricarboxylic acid (TCA) cycle of *T. asperellum* TJ01 (Fig. 3a).

KEGG analysis based on RNA-seq of *T. asperellum* TJ01 with 500 μg/mL dichlorvos treatment showed that 189 genes related to amino acid metabolism, 93 genes related to lipid metabolism, and 60 genes related to carbohydrate metabolism were significantly altered by dichlorvos. LC-QQQ-MS and GC-TOF-MS analysis of *T. asperellum* TJ01 treated with 500 μg/mL dichlorvos showed that, compared with the control, 16 (38%) amino acids and derivatives were significantly upregulated, while 26 (62%) were significantly downregulated (Fig. 3b; Additional file 2). Moreover, seven (39%) carbohydrates were significantly upregulated and 11 (61%) were significantly downregulated (Fig. 3c; Additional file 3), while 39 (89%) lipids were significantly upregulated and five (11%) were significantly downregulated (Fig. 3d; Additional file 4).

### Impact of dichlorvos on secondary metabolism of *T. asperellum* TJ01

**Fig. 4:**
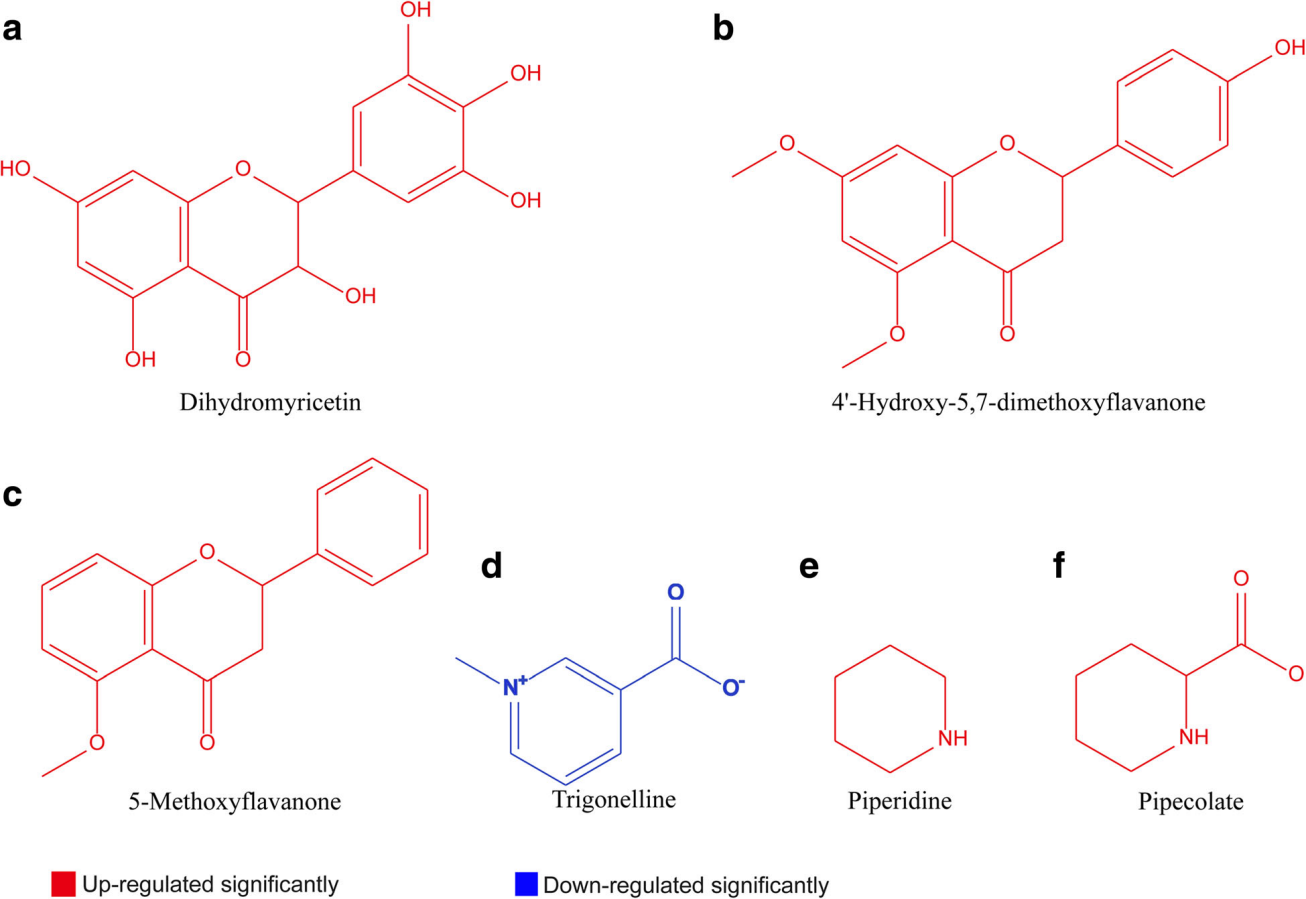
Impact of dichlorvos on secondary metabolism of *T. asperellum* TJ01 based on GC-TOF-MS and LC-QQQ-MS analysis. **a**, Dihydromyricetin; **b**, 4'-hydroxy-5,7-dimethoxyflavanone; **c**, 5-methoxyflavanone; **d**, Trigonelline; **e**, Piperidine; **f**, Pipecolate. Red: significantly upregulated, blue: significantly downregulated

In *T. asperellum* TJ01 treated with 100 μg/mL, 500 μg/mL, and 1000 μg/mL dichlorvos, 32, 81, and 67 genes about secondary metabolism, respectively, were significantly altered based on RNA-seq analysis (Additional file 5). LC-QQQ-MS and GC-TOF-MS analysis of *T. asperellum* TJ01 treated with 500 μg/mL dichlorvos showed that, compared with the control, four flavonoids (5-methoxyflavanone, dihydromyricetin, afzelechin, and 4′-hydroxy-5,7-dimethoxyflavanone), one flavonol [isorhamnetin (3′-methoxyquercetin)], and three alkaloids (piperidine, pipecolate, and galactinol) were significantly upregulated, while one alkaloid (trigonelline) was significantly downregulated (Fig. 4; Additional file 6). In addition to these, many other secondary metabolites were significantly altered, such as griseofulvin, carnitine, and hycanthone.

### Impacts of dichlorvos on *T. asperellum* TJ01 transcription factors and stress resistance genes

**Fig. 5:**
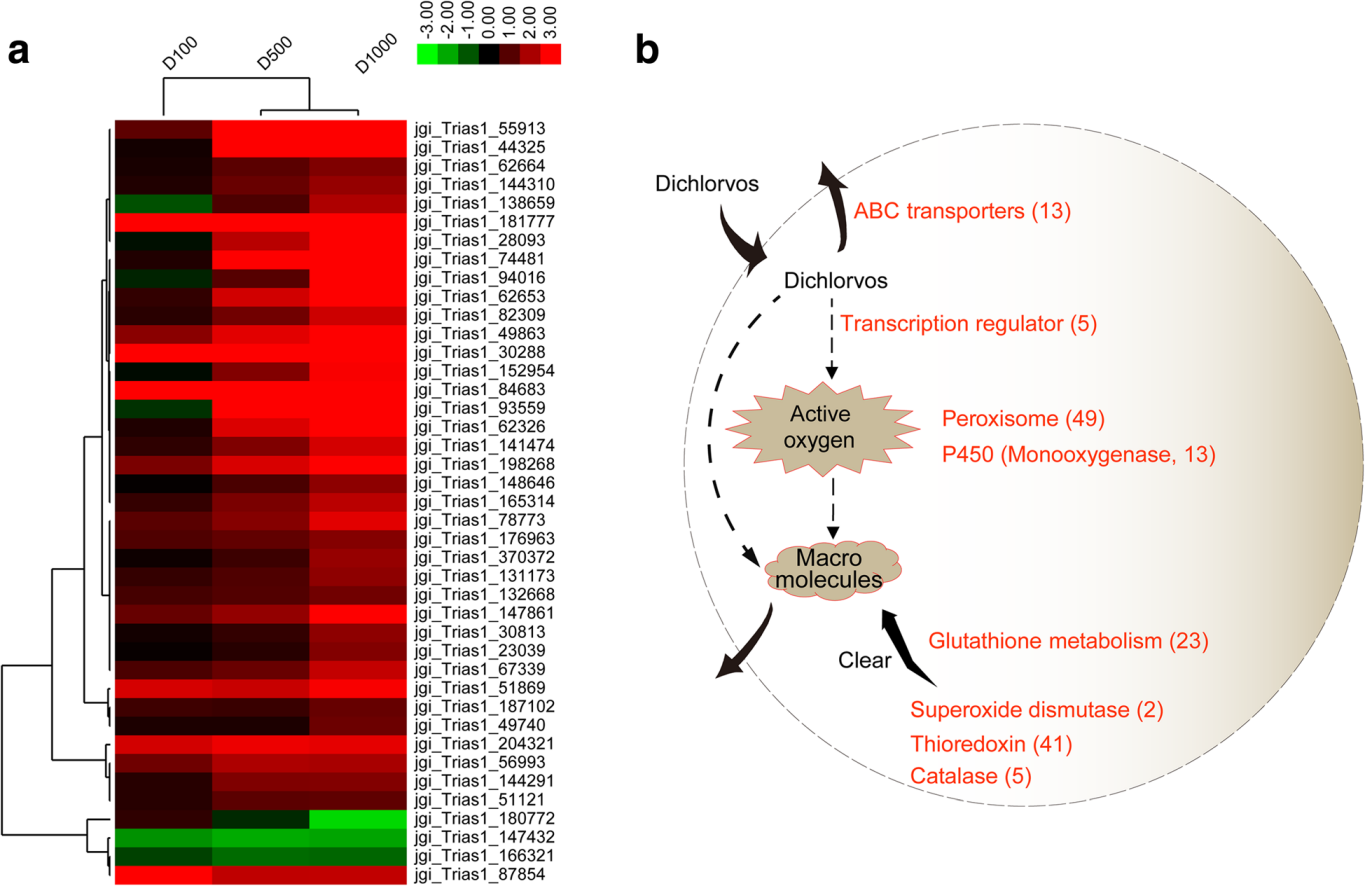
Speculated mechanism of *T. asperellum* TJ01 resistance to dichlorvos. **a** Expression analysis of thioredoxin in *T. asperellum* TJ01 under the stress of different concentrations of dichlorvos. D100: log_2_ 100 μg/mL dichlorvos treatment/CK, D500: log_2_ 500 μg/mL dichlorvos treatment/CK, D1000: log_2_ 1000 μg/mL dichlorvos treatment/CK; **b** Diagram of *T. asperellum* TJ01 resistance to dichlorvos. Red words represent significantly upregulated genes and their number

In order to reveal the mechanisms of *T. asperellum* TJ01 tolerance to dichlorvos, detoxification- and tolerance-related genes were analyzed. Comparing with control, after 24 h of treatment with dichlorvos, five transcriptional regulators were significantly upregulated (Table 1). Meanwhile, a large number of redox proteins were significantly upregulated, including 49 peroxidases, 13 P450 monooxygenases, 23 glutathione metabolism proteins, two superoxide dismutases, five catalases, and 41 thioredoxins (Fig. 5; Additional file 7).

**Table 1:**
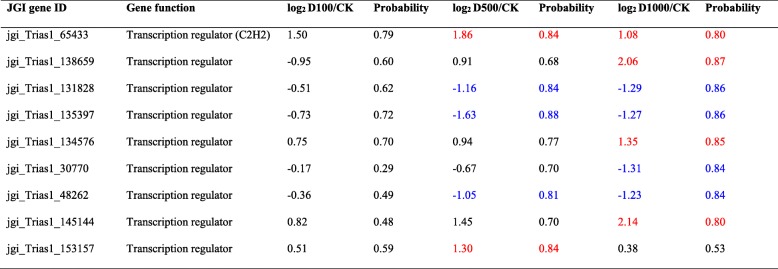
Impact of dichlorvos with different concentrations on transcription regulator of *T. asperellum* TJ01 after 24 h treatment based on RNA-seq

Red represents up-regulated significantly, while blue represents down-regulated significantly

Additionally, after 24 h of treatment with dichlorvos, 13 ABC transporter genes were found to be significantly upregulated (Fig. 5b; Additional file 7).

### Impact of dichlorvos on target genes of *T. asperellum* TJ01

Acetylcholinesterase (AChE) is a key enzyme that terminates nerve impulses by catalyzing the hydrolysis of the neurotransmitter acetylcholine in the nervous system. RNA-seq results showed that there were a total of 16 acetylcholinesterase genes in *T. asperellum* TJ01. Comparing with control, none were significantly altered following dichlorvos treatment, except for one under 1000 μg/mL dichlorvos (Table 2).

**Table 2:**
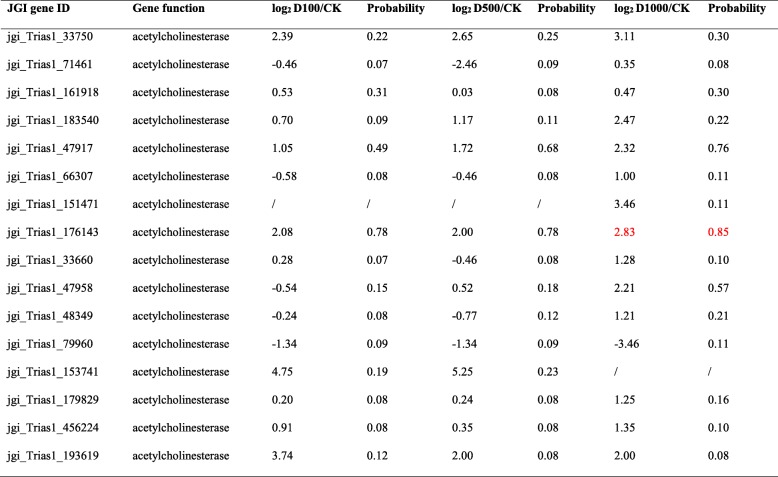
Impact of dichlorvos with different concentrations on acetylcholinesterase of *T. asperellum* TJ01 based after 24 h treatment on RNA-seq

Red represents up-regulated significantly

### Absorption and residue degradation of dichlorvos by *T. asperellum* TJ01

**Fig. 6:**
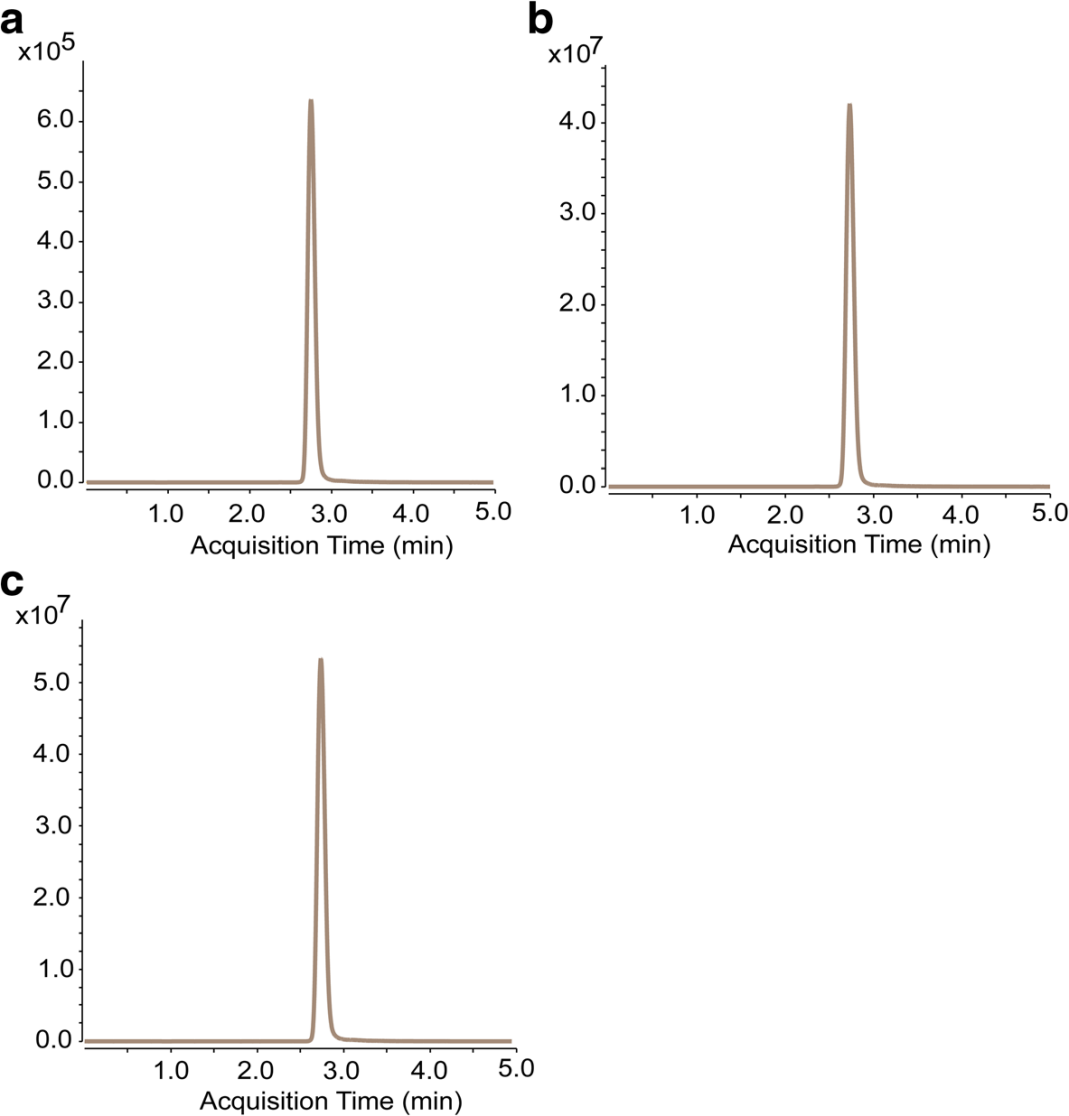
Absorption and residue degradation of dichlorvos after co-fermentation with *T. asperellum* TJ01 for 3 d according to UHPLC-MS/MS detection. **a** Dichlorvos absorbed into *T. asperellum* TJ01; **b** Residual dichlorvos in broth after co-fermentation with *T. asperellum* TJ01; **c** Dichlorvos in broth following pure fermentation

UHPLC-MRM-MS/MS results showed that, after co-fermentation of *T. asperellum* TJ01 and 500 μg/mL dichlorvos in Burk broth for 3 d, the content of dichlorvos in *T. asperellum* TJ01 was 0.015 μg/mL (Fig. 6a) and the content of dichlorvos in the broth was 322.663 μg/mL (Fig. 6b). In contrast, after the pure fermentation of 500 μg/mL dichlorvos in Burk broth for 3 d, the content of dichlorvos was 415.908 μg/mL (Fig. 6c), while the self-degradation of dichlorvos was 84.092 μg/mL. This shows that *T. asperellum* TJ01 has a limited capacity to absorb dichlorvos, as most of the dichlorvos was still present in the fermentation broth.

## Discussion

*Trichoderma* is one of the most widely used fungi in the field of biological control, as it exerts high activity and is environmentally friendly [11, 12]. However, due to the fact that it is slow-acting and has a short shelf life, in actual production, *Trichoderma* still cannot completely replace traditional chemical pesticides. At present, the most ideal control strategy may be a combination of *Trichoderma* and chemical pesticides, which would reduce the amount of pesticides used and achieve sustained control. Therefore, the tolerance of *Trichoderma* to dichlorvos is particularly critical. In previous work, it was found that *T. atroviride* had a good tolerance to dichlorvos [13], but there are no reports of the tolerance of *T. asperellum.* Recently, we isolated *T. asperellum* TJ01, which not only has a fast growth rate (strong competition), but also has high tolerance to dichlorvos. So this strain was chose as an in-depth study, hoping to lay a certain theoretical foundation for the production of complex microbial pesticides in agriculture.

There have been no thorough and comprehensive studies on the related mechanisms of *Trichoderma* tolerance to dichlorvos. Therefore, revealing the specific molecular mechanisms has become particularly important. In this study, transcriptome and metabolome data were used to analyze the mechanism underlying the response of *Trichoderma asperellum* TJ01 to dichlorvos. This represents the first detailed report of the global effects of dichlorvos on *Trichoderma*.

Primary metabolism is the process by which microorganisms absorb various nutrients from the outside world and produce energy and substances, including synthetic sugars, amino acids, common fatty acids, and nucleic acids, to sustain life activities through decomposition and anabolism. The TCA cycle is the basis of biological metabolism and is the hub of the carbohydrate, fatty acid, and amino acid metabolism pathways [14]. In this study, comprehensive analysis of transcriptome and metabolome data suggested that dichlorvos has obvious effect on primary metabolism of *T. asperellum* TJ01 (Fig. 3) [15]. Among the significantly upregulated lipid compounds, most were lysophosphatidylethanolamines and lysophosphatidylcholines. According to reports, most lysophosphatidylethanolamines and lysophosphatidylcholines are related to stress resistance, including LysoPC (lysophosphatidylcholine, 14:0), LysoPC (15:0), LysoPC (18:1), LysoPC (20:2), and LysoPC (20:1) (Additional file 4) [16, 17].

Secondary metabolism refers to the biosynthesis of non-essential substances and the storage of secondary metabolites. *Trichoderma* spp. secrete many secondary metabolites [18]. Some of these secondary metabolites are related to pathogen inhibition [19], while some are involved in stress resistance, such as flavonoids and alkaloids. Flavonoids act as a secondary antioxidant defense system in plant tissues exposed to different abiotic and biotic stresses [20]. It has also been reported that plant alkaloids may be impacted by drought stress and soil pH [21, 22]*.* However, there have been no similar reports in fungi and bacteria. In this study, four flavonoids and one alkaloid were significantly upregulated under the stress of dichlorvos (Fig. 4 and Additional file 6).

Transcription factors are important players in signal transduction pathways and determine the function of cells in many ways. There are 12 superfamily and 37 Protein family (PFAM) families containing transcription factor DNA binding domains in most fungal species [23, 24]. C_2_H_2_ zinc finger proteins are a large class of transcription factors in fungi, with 70–80 C_2_H_2_ zinc finger protein genes predicted in *Trichoderma*. It has been experimentally confirmed that *carbon catabolite repression 1* (*cre1*) is involved in carbon metabolism [25], *cseb1* is involved in response to osmotic stress, and *Blue-light 7* (*blu7*) is involved in tolerance to light responsiveness [26]; however, a large number of genes remain to be characterized. In *T. asperellum* TJ01 treated with different concentrations of dichlorvos, a novel C_2_H_2_ zinc lipoprotein transcriptional regulator that may be involved in stress resistance (Table 1). Additionally, in order to reveal the mechanisms of *T. asperellum* TJ01 tolerance to dichlorvos, detoxification- and tolerance-related genes were analyzed [27, 28]. After dichlorvos treatment, a large number of redox proteins were significantly upregulated. This confirms that the ability of *Trichoderma* to withstand relatively high concentrations of a variety of synthetic and natural toxic compounds, including its own antibiotics, depends on efficient cell detoxification mechanisms, supported by a complex system of membrane pumps. It is well known that the genome of *Trichoderma* includes ABC transporters, which are members of a protein superfamily involved in the efflux of drugs from the cells of target organisms. These ABC transporters may explain the natural tolerance of *Trichoderma* to fungicides and their ability to successfully survive in extreme environments [28–30]. After dichlorvos treatment, lots of ABC transporter genes were found to be significantly upregulated. We speculate that all of the factors mentioned above contribute to the tolerance mechanism of *T. asperellum* TJ01 to dichlorvos (Fig. 5).

AChE is a key enzyme that terminates nerve impulses by catalyzing the hydrolysis of the neurotransmitter acetylcholine in the nervous system. Organophosphorus insecticides, such as dichlorvos, target AChE and irreversibly inhibit the enzyme by phosphorylating a serine hydroxyl group within the enzyme active site [31]. In this study, nearly none predicted *AChE* genes were significantly altered following dichlorvos treatment (Table 2). This may also be another reason for the fact that dichlorvos and *T. asperellum* TJ01 are able to co-exist at certain concentrations.

It has been reported that *Trichoderma* has a high tolerance and biosorption capacity of heavy metals like Zn^2+^, Pb^2+^, Ni^3+^, and Cu^2+^ [32]. However, the adsorption rate of dichlorvos by *Trichoderma* has not been reported. UHPLC-MRM-MS/MS results showed that *T. asperellum* TJ01 has a limited capacity to absorb dichlorvos. Interestingly, compared with the control, 93.23 μg/mL dichlorvos was missing from both the mycelium and the fermentation solution (Fig. 6). We speculated that this is being degraded by *T. asperellum* TJ01. It has already been reported that *T. atroviride* has the ability to degrade dichlorvos [33, 34]. Details of the degradation product of dichlorvos following degradation by *T. asperellum* TJ01 should be investigated in future studies.

This study provided a strong theoretical basis for the combination of dichlorvos and *T. asperellum* TJ01, which could be implemented in several ways, for example, dichlorvos with biocontrol factors or spores granules of *T. asperellum* TJ01. Therefore, future studies should evaluate the interaction of *T. asperellum* and dichlorvos in the presence of pathogens and plants.

## Conclusions

In this study, association analyses of RNA-seq, GC-TOF-MS, and LC-QQQ-MS data revealed the global primary and secondary metabolic changes of *T. asperellum* TJ01 under dichlorvos stress. In addition, a novel C_2_H_2_ zinc finger protein gene, *zfc1,* was discovered and found to be upregulated in the presence of dichlorvos, along with a large number of oxidoreductase and ABC transporter genes, and then the tolerance mechanism of *T. asperellum* TJ01 to dichlorvos was proposed. Finally, the absorption and residue of dichlorvos were analyzed by LC-QQQ-MS, laying the foundation for the use of *T. asperellum* TJ01 in the degradation of pesticide residues.

## Methods

### Strain and reagent

The biocontrol fungus *T. asperellum* TJ01 was isolated from irrigated soil sample was collected in Fuyang, Anhui, China and stored at the School of Biotechnology and Food Engineering, Fuyang Normal College, Fuyang, Anhui, China. An analytical-grade standard of dichlorvos was purchased from the Shanghai Pesticide Research Institute Company.

### Growth of *T. asperellum* TJ01 under dichlorvos stress

*T. asperellum* TJ01 was cultured in PDA dishes for 3 d, after which holes were punched in the *T. asperellum* TJ01 culture dish with a 7-mm punch. The dish was then turned upside-down over a fresh PDA or Burk (more suitable for studying mechanism as the clean background) plate [35] containing 0 μg/mL (control), 100 μg/mL, 500 μg/mL, or 1000 μg/mL dichlorvos. Plates were incubated at 28 °C for 3 d. The overall growth and sporulation of *T. asperellum* TJ01 were observed and photographed each day.

### Effect of dichlorvos on transcription of *T. asperellum* TJ01 as determined by RNA-seq

The spore suspension of *T. asperellum* TJ01 was transferred to PD medium. After culturing at 28 °C and 180 rpm for 24 h, 2 g of mycelia was collected by vacuum filtration and transferred to Burk broth containing 0 μg/mL (control), 100 μg/mL, 500 μg/mL, or 1000 μg/mL dichlorvos. After culturing at 28 °C and 180 rpm for 24 h, the mycelia were collected by vacuum filtration and frozen with liquid nitrogen. RNA extraction, cDNA library construction, and Solexa sequencing were performed at the Beijing Genomics Institute (BGI, Beijing, China). Each experiment was repeated three times.

According to the reference genome (downloaded from http://genome.jgi.doe.gov/Trias1/Trias1.download.html) [36], conventional transcriptome analyses were performed, including GO (Gene Ontology) /KEGG (Kyoto Encyclopedia of Genes and Genomes) enrichment analysis of significantly differentially expressed genes [37, 38]. The NOISeq method was used to screen differently expressed miRNAs between two groups [39]. A log_2_(fold change) > 1 and probability > 0.8 indicated genes that were significantly upregulated, while a log_2_(fold change) < − 1 and probability > 0.8 indicated genes that were significantly downregulated.

Specific analyses of primary and secondary metabolic pathways were performed using the JGI website (https://genome.jgi.doe.gov/portal/).

### Impact of dichlorvos on metabolome of *T. asperellum* TJ01 as determined by GC-TOF-MS analysis

The spore suspension of *T. asperellum* TJ01 was transferred to PD medium. After culturing at 28 °C and 180 rpm for 24 h, 2 g of mycelia was collected by vacuum filtration and transferred to Burk broth containing 0 μg/mL (control) or 500 μg/mL dichlorvos. After culturing at 28 °C and 180 rpm for 24 h, the mycelia were collected by vacuum filtration and frozen with liquid nitrogen.

GC-TOF-MS sample processing was performed as previously described [40]. GC-TOF-MS analysis was performed using an Agilent 7890 gas chromatography system coupled with a Pegasus HT time-of-flight mass spectrometer.

The system was equipped as previously described [40]. A 1-μL aliquot of the analyte was injected in splitless mode. Helium was used as the carrier gas, and the gas flow rate through the column was 1 mL min^− 1^. The initial temperature was kept at 50 °C for 1 min, then raised to 310 °C at a rate of 10 °C min^− 1^, then maintained at 310 °C for 8 min. The injection, transfer line, and ion source temperatures were 280, 270, and 220 °C, respectively. The energy was − 70 eV in electron impact mode. The mass spectrometry data were acquired in full-scan mode with an *m/z* range of 50–500 at a rate of 20 spectra per second after a solvent delay of 6.1 min [41, 42].

Chroma TOF 4.3X software (LECO Corporation) and the LECO-Fiehn Rtx5 database were used for raw peak exaction, baseline filtering and calibration, peak alignment, deconvolution analysis, peak identification, and integration of the peak area. Metabolic features detected in < 50% of QC samples were removed [42].

### Global impact of dichlorvos on *T. asperellum* TJ01 metabolome as determined by LC-QQQ-MS analysis

The spore suspension of *T. asperellum* TJ01 was transferred to PD medium. After culturing at 28 °C and 180 rpm for 24 h, 2 g of mycelia was collected by vacuum filtration and transferred to Burk broth containing 0 μg/mL (control) or 500 μg/mL dichlorvos. After culturing at 28 °C and 180 rpm for 24 h, the mycelia were collected by vacuum filtration and frozen with liquid nitrogen.

The sample processing was performed as previously describeds [43]. Then the sample extracts were analyzed using an LC-ESI-MS/MS system (HPLC, Shim-pack UFLC SHIMADZU CBM30A system, www.shimadzu.com.cn/; MS, Applied Biosystems 4500 Q TRAP, https://sciex.com/products/mass-spectrometers/qtrap-systems/qtrap-4500-system). The analytical conditions for HPLC were as previously described [44], except that the column was that of Waters ACQUITY UPLC HSS T3 C18 (1.8 μm, 2.1 mm × 100 mm) and the gradient program was modified, 100:0 *V*/V at 0 min, 5:95 V/V at 11.0 min, 5:95 V/V at 12.0 min, 95:5 V/V at 12.1 min, 95:5 V/V at 15.0 min; flow rate, 0.40 mL/min. QQQ scans were acquired as MRM experiments and a specific set of MRM transitions were monitored for each period according to the metabolites eluted within this period [45].

Based on the self-built Metware database (MWDB) and the public database of metabolite information, qualitative analysis of the first-order and second-order spectra detected by mass spectrometry were performed. Some of substances were qualitatively analyzed by removing isotopic signals; repetitive signals containing K^+^, Na^+^, and NH4^+^ ions; and repeated signals of fragmented ions that themselves are of larger molecular weight. Metabolite structural elucidation was performed in reference to MassBank (https://massbank.eu/MassBank/), KNAPSAcK (http://kanaya.naist.jp/KNApSAcK/), HMDB (http://www.hmdb.ca/), and existing mass spectrometry public databases such as MoTo DB (http://www.ab.wur.nl/moto/) and METLIN (http://metlin.scripps.edu/index.php) [46].

### Degradation and absorption of dichlorvos by *T. asperellum* TJ01 as determined by UHPLC-MS/MS

*T. asperellum* TJ01 spore suspension was transferred to PD medium to a final spore concentration of 10^6^ cfu/mL. After culturing at 28 °C and 180 rpm for 2 d, 2 g of seed mycelium was transferred to Burk broth containing 500 μg/mL dichlorvos. After culturing at 28 °C and 180 rpm for 3 d, the culture was filtered through a 0.22-μm membrane filter. The mycelia of *T. asperellum* TJ01 were collected by vacuum filtration and washed twice with 4 °C sterile deionized water. Filtered Burk broth containing 500 μg/mL dichlorvos was used as a control.

For the liquid sample, a 10-μL aliquot of the sample was transferred to an EP tube with 990 μL of extraction solvent (acetonitrile:methanol:water, 2:2:1, precooled to − 20 °C). The mixture was vortexed for 30 s and centrifuged for 15 min at 12000 rpm and 4 °C. Then, the supernatant was transferred to an auto-sampler vial for UHPLC-MS/MS analysis (dilution factor = 100).

For the mycelim sample, 161.8 mg was precisely weighed and transferred to an Eppendorf tube with 1000 μL of extraction solvent (acetonitrile:methanol:water, 2:2:1, precooled to − 20 °C). The mixture was vortexed for 30 s, homogenized for 4 min at 45 Hz, and sonicated for 5 min in an ice-water bath. Homogenization and sonication were repeated three times, followed by incubation at − 20 °C for 1 h and centrifugation for 15 min at 12000 rpm and 4 °C. An 80-μL aliquot of the supernatant was transferred to an auto-sampler vial for LC-MS/MS analysis.

## Additional files

Additional file 1: Impact of dichlorvos with different concentrations on TCA cycle of *T. asperellum* TJ01 after 24 h treatment based on RNA-seq. Red represents up-regulated significantly, while blue represents down-regulated significantly. (XLS 7 kb)

Additional file 2: Impact of 500 μg/mL dichlorvos on amino acid and derivatives metabolism of *T. asperellum* TJ01 after 24 h treatment based on LC-QQQ-MS and GC-TOF-MS analysis. Red represents up-regulated significantly, while blue represents down-regulated significantly. (XLS 11 kb)

Additional file 3: Impact of 500 μg/mL dichlorvos on carbohydrate metabolism of *T. asperellum* TJ01 after 24 h treatment based on LC-QQQ-MS and GC-TOF-MS analysis. Red represents up-regulated significantly, while blue represents down-regulated significantly. (XLS 9 kb)

Additional file 4: Impact of 500 μg/mL dichlorvos on lipid metabolism of *T. asperellum* TJ01 after 24 h treatment based on LC-QQQ-MS and GC-TOF-MS analysis. Red represents up-regulated significantly, while blue represents down-regulated significantly. (XLS 11 kb)

Additional file 5: Number of significantly differentially expressed genes of secondary metabolism based on RNA-seq analysis of *T. asperellum* TJ01 after treatment with dichlorvos for 24 h. D100: 100 μg/mL dichlorvos treatment/CK; D500: 500 μg/mL dichlorvos treatment/CK; D1000: 1000 μg/mL dichlorvos treatment/CK. (TIF 504 kb)

Additional file 6: Impact of 500 μg/mL dichlorvos on flavonoids and alkaloids of *T. asperellum* TJ01 after 24 h treatments based on LC-QQQ-MS and GC-TOF-MS analysis. Red represents up-regulated significantly, while blue represents down-regulated significantly. (XLS 9 kb)

Additional file 7: Impact of dichlorvos with different concentrations on detoxification/tolerance mechanism related genes of *T. asperellum* TJ01 after 24 h treatment based on RNA-seq. Red represents up-regulated significantly, while blue represents down-regulated significantly. (XLS 24 kb)

## Abbreviations

ABC transporter: ATP binding cassette transporter
AChE: Acetylcholinesterase
ATP: Adenosine triphosphate
AtrR: ABC-transporter regulating transcription factor
BGI: Beijing Genomics Institute
blu7: Blue-light 7
cre1: Carbon catabolite repression
cyp51A: Cytochrome P450 Family 51 Subfamily A
GC-TOF-MS: Gas chromatography-mass spectrometry
GO: Gene Ontology
KEGG: Kyoto Encyclopedia of Genes and Genomes
LC-QQQ-MS: Liquid chromatography-mass spectrometry
LysoPC: Lysophosphatidylcholine
PDA: Potato Dextrose Aga
Pdr1: Pleiotropic drug resistance 1
PFAM: Protein family
TCA: Tricarboxylic acid
UHPLC: Ultra-high performance liquid chromatography
zfc1: zinc finger chimera 1
