# Supplementary figures and images for: Omics for understanding the tolerant mechanism of Trichoderma asperellum TJ01 to organophosphorus pesticide dichlorvos
Source: BMC Genomics. 2018 Aug 8;19:596. doi: 10.1186/s12864-018-4960-y (PMC6083568; doi:10.1186/s12864-018-4960-y)

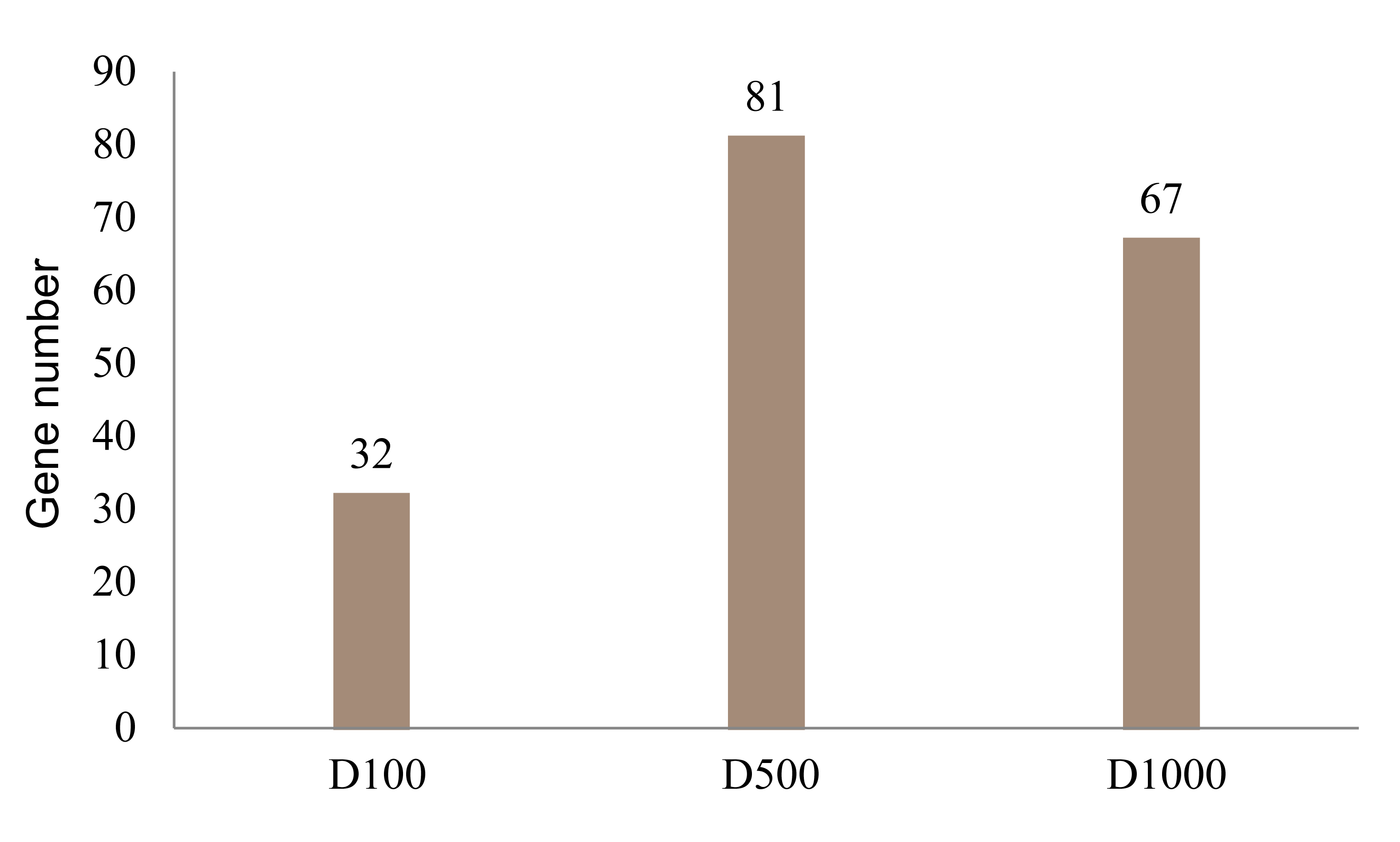

Supplement: Supplementary file 5 — Number of significantly differentially expressed genes of secondary metabolism based on RNA-seq analysis of T. asperellum TJ01 after treatment with dichlorvos for 24 h. D100: 100 μg/mL dichlorvos treatment/CK; D500: 500 μg/mL dichlorvos treatment/CK; D1000: 1000 μg/mL dichlorvos treatment/CK. (TIF 504 kb) [file 12864_2018_4960_MOESM5_ESM.tif]
